# Deadly acceleration in dehydration of *Eucalyptus viminalis* leaves coincides with high-order vein cavitation

**DOI:** 10.1093/plphys/kiad016

**Published:** 2023-01-24

**Authors:** Vanessa Tonet, Madeline Carins-Murphy, Ross Deans, Timothy J Brodribb

**Affiliations:** School of Biological Sciences, University of Tasmania, Sandy Bay, Tasmania 7001, Australia; School of Biological Sciences, University of Tasmania, Sandy Bay, Tasmania 7001, Australia; ARC Centre of Excellence in Translational Photosynthesis, Division of Plant Science, Research School of Biology, The Australian National University, Canberra, Australian Capital Territory 2601, Australia; School of Biological Sciences, University of Tasmania, Sandy Bay, Tasmania 7001, Australia

## Abstract

Xylem cavitation during drought is proposed as a major driver of canopy collapse, but the mechanistic link between hydraulic failure and leaf damage in trees is still uncertain. Here, we used the tree species manna gum (*Eucalyptus viminalis*) to explore the connection between xylem dysfunction and lethal desiccation in leaves. Cavitation damage to leaf xylem could theoretically trigger lethal desiccation of tissues by severing water supply under scenarios such as runaway xylem cavitation, or the local failure of terminal parts of the leaf vein network. To investigate the role of xylem failure in leaf death, we compared the timing of damage to the photosynthetic machinery (*F*_v_/*F*_m_ decline) with changes in plant hydration and xylem cavitation during imposed water stress. The water potential at which *F*_v_/*F*_m_ was observed to decline corresponded to the water potential marking a transition from slow to very rapid tissue dehydration. Both events also occurred simultaneously with the initiation of cavitation in leaf high-order veins (HOV, veins from the third order above) and the analytically derived point of leaf runaway hydraulic failure. The close synchrony between xylem dysfunction and the photosynthetic damage strongly points to water supply disruption as the trigger for desiccation of leaves in this hardy evergreen tree. These results indicate that runaway cavitation, possibly triggered by HOV network failure, is the tipping agent determining the vulnerability of *E. viminalis* leaves to damage during drought and suggest that HOV cavitation and runaway hydraulic failure may play a general role in determining canopy damage in plants.

## Teaser

Vein network failure and runaway cavitation represent the critical threshold beyond which leaves are critically damaged and die.

## Introduction

Extensive canopy collapse has been observed worldwide and linked to enhanced and protracted drought conditions associated with changing rainfall patterns and increasing temperatures (Lloret et al., 2004; Allen et al., 2010; Matusick et al., 2013; Nardini et al., 2013; Li et al., 2018). Drought events pose risks to leaves, with major implications for net primary productivity, carbon balance, and biodiversity (Anderegg et al., 2013). However, even under the simplest scenario of acute drought, the cause of leaf death in trees is unresolved and not explicitly linked to any physiological mechanism. For predicting the extent of canopy mortality and tree die-off in future droughts, we need to understand the physiological triggers of leaf death.

Leaf hydration is maintained by water transported from the soil through the plant water transport system (xylem) under a water potential gradient from the soil to the atmosphere. During water shortage, the closure of stomatal pores reduces water loss from leaves, but if the soil continues to dry and drought stress increases, the water potential in the xylem continues to fall. In a process that remains incompletely understood (Pereira et al., 2022), the xylem becomes damaged once water potentials fall to a point where tiny air bubbles break into the water column and rapidly expand in a process historically described as xylem cavitation (Tyree and Sperry, 1989). Since the discovery of cavitation-induced damage to the xylem during water stress, different thresholds have been proposed to predict the limits of tree survival. In terms of xylem damage thresholds, the water potential corresponding to the loss of 50% of stem water transport capacity (P_50_) has been traditionally used as an index of drought vulnerability (Johnson et al., 2012; Anderegg et al., 2016) to characterize and compare different plant species. Yet despite the use of stem cavitation thresholds to predict mortality (Brodribb and Cochard, 2009; Urli et al., 2013; Blackman et al., 2014; Nardini and Luglio, 2014; Anderegg, 2015), there is no clear evidence why a partial loss of hydraulic capacity in the stem should cause canopy dieback (Savi et al., 2016) considering the very substantial hydraulic capacity that remains even after 50% of the stem xylem is cavitated (Dietrich et al., 2018).

To predict the conditions that trigger leaf death, it is important to consider the connected mechanisms involved in the process. During an acute episode of drought, continued transpiration (albeit greatly diminished by stomatal closure) ensures that leaves remain at the most negative end of the water potential spectrum in plants (Elfving et al., 1972; Passioura, 1982; Tyree and Sperry, 1989; Sperry et al., 2003; McDowell et al., 2008). This rate of dehydration is buffered by water flowing into the leaf through the xylem from either the soil (assuming a small connection remains between the roots and soil) or capacitance of connected tissues. Thus, damage to the distal part of the water transport system (the leaf veins) has the potential to greatly accelerate leaf tissue dehydration. In the same way that turning the tap off will kill plants in an irrigated Saudi Arabian corn field, major cavitation in the leaves has the potential to cause cell death in downstream mesophyll tissue, regardless of the specific resilience of those particular cells (Porembski, 2011).

Hydraulic failure (here intended as the complete loss of hydraulic conductance to or within an organ) can most easily be theorized as a mortality agent following the “runaway cavitation” hypothesis. Tyree and Sperry (1988) described this as a feedback cycle in which the blockage of the xylem by embolism reduces the hydraulic conductance, causing water potential to decline in tissue downstream of the blockage, leading to more cavitation and so on, until the point of hydraulic failure. Under normal circumstances runaway cavitation would be expected to occur when there was a high percentage of embolism in the tissue (>90%), such that even a small amount of water loss caused by transpiration would decrease the water potential, thus initiating this positive feedback. For leaves, the disconnection from the stem or soil water supply causes tissues to rapidly dehydrate and die from desiccation (Nardini et al., 2013; Brodribb et al., 2016; Cardoso et al., 2020; Brodribb et al., 2021; Chen et al., 2021). The few studies that have focused on this vicious cycle (Tyree and Sperry, 1988; Mencuccini and Comstock, 1997; Nardini and Salleo, 2000) report it as a theoretical explanation rather than providing empirical evidence of its connection to tissue damage during drought.

Providing evidence to connect dynamic processes of vascular and tree damage is hugely challenging due to the existence of different lags and thresholds that may temporally disconnect cause and effect in the process of tree mortality. The leaf however provides a much more tractable scale. Leaves are generally observed to be the most vulnerable tissues to dehydration damage (Choat et al., 2005; Hochberg et al., 2016; Sack et al., 2016). At the same time, they offer the greatest possibility to explore the processes that may connect damage to the water transport system to the death of living tissues. The function of the leaf water transport system is more complicated than the stem due to the particular arrangement of its xylem in veins of different orders, from the midrib (departing from the lamina base to the leaf apex) to the second order (branching from the midrib, and generally referred as major veins) to the higher order (consisting of small veins forming a reticulate system that irrigates the mesophyll) (Sack and Scoffoni, 2013; Gleason et al., 2018). This two-dimensional system consists typically of numerous anastomoses that complicate our understanding of how cavitation affects leaf water transport (Nardini et al., 2003; Brodribb et al., 2016). Despite the leaf vein system being characterized by network redundancy in the sense that flow restrictions due to damage in larger veins can be overcome by interconnections that allow water to bypass blockages (Plymale and Wylie, 1944), the likelihood remains that damage to smaller veins may isolate local regions of the leaf (Brodribb et al., 2021).

**Figure 1:**
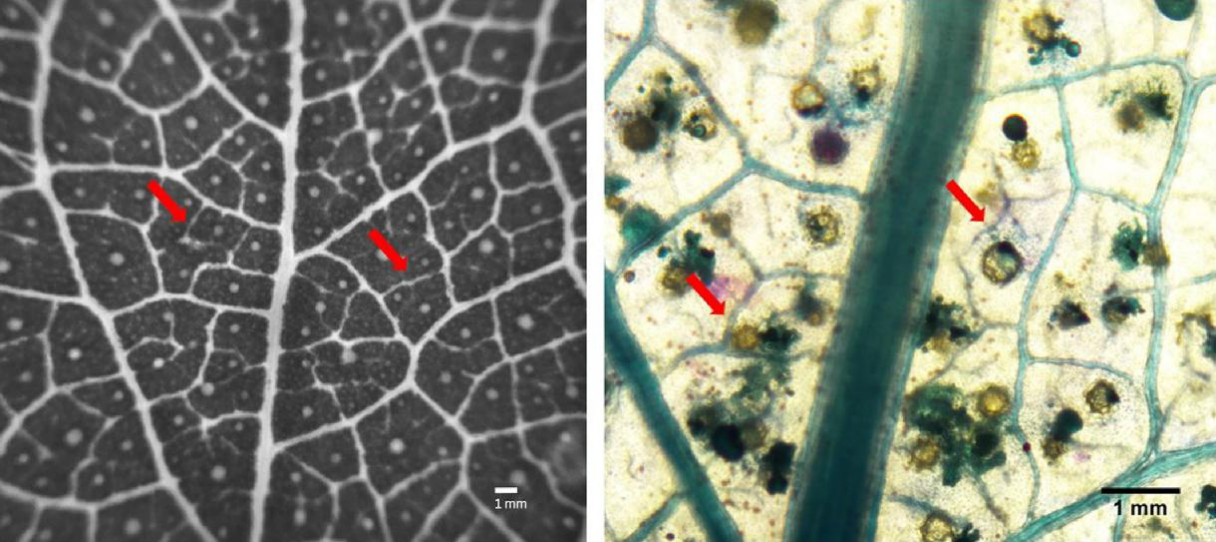
Vein network of *E. viminalis* leaves. A, Representative raw image of *E. viminalis* leaf vasculature captured using the OVT. B, Light micrograph of the leaf vasculature at 4× magnification. Arrows on both images indicate HOV.

Here we hypothesized that the tipping agent in leaf desiccation is the occurrence of runaway cavitation culminating in the failure of HOV (high order veins, veins from the third order above) to deliver water to the mesophyll. We selected the tree species *Eucalyptus viminalis* Labill. given its ecological importance and a recent record of extensive dieback (Ross and Brack, 2017). We track the relationship between xylem embolism formation, leaf hydration, and photosynthetic activity in the leaves of whole saplings to determine if runaway cavitation or other metrics of plant damage (turgor loss point, leaf vein cavitation, or stem cavitation) were linked to the lethal dehydration of leaves. We were able to calculate the magnitude of hydraulic loss that would trigger runaway cavitation and compare this with the point of tissue damage, thus allowing us to determine the mechanism of hydraulic failure in leaves of *E. viminalis* (Figure 1).

## Results

### Changes to fluorescence and leaf width with time and drought stress

**Figure 2:**
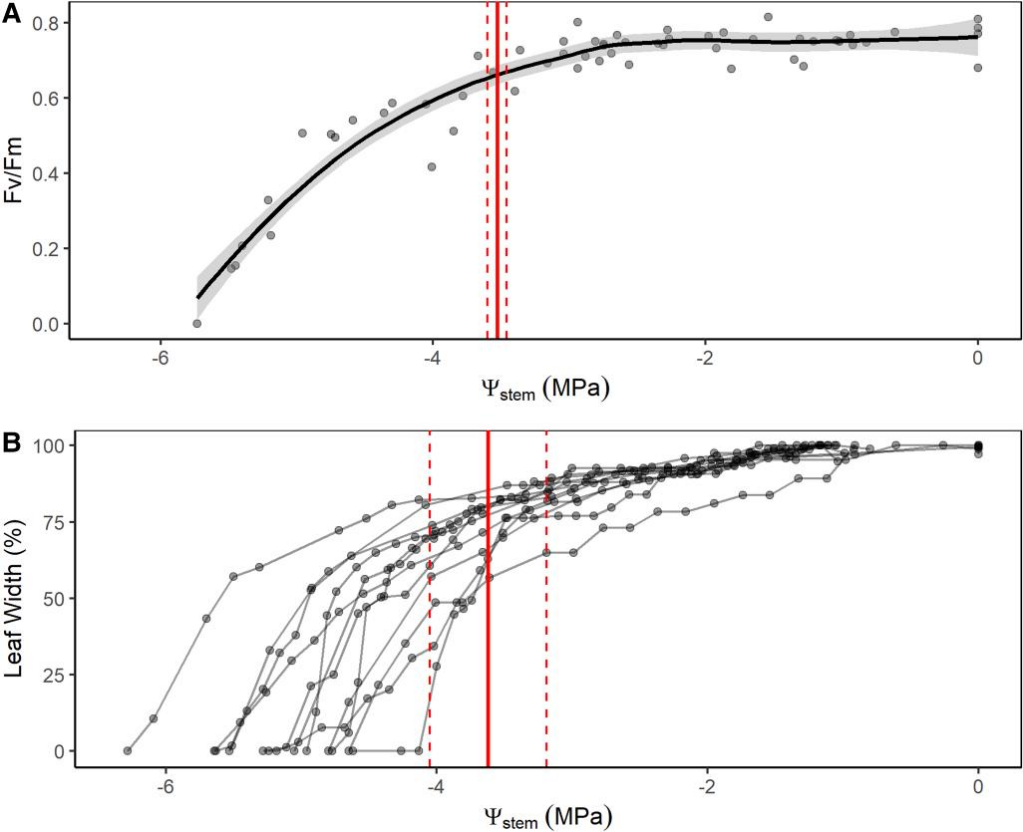
Fluorescence and leaf width dynamic during dehydration. Changes to leaf fluorescence (*F*_v_/*F*_m_) (A) and width (B) with declining stem water potential (Ψ_stem_, MPa). Solid black line in plot (A) represents a regression with 95% confidence intervals (light gray shading). Red solid lines represent the mean water potential at the slope breakpoint in *F*_v_/*F*_m_ and leaf width, respectively, and dashed lines represent the Sd (standard deviation).

The dehydration of whole plants was characterized by an initial period of static leaf *F_v_*/*F_m_* accompanying a slow decline in leaf width over 2–3 days. This initial slow period of change was followed by a sudden transition to rapidly declining leaf width alongside a rapid decline in *F_v_*/*F_m_* in days 5 and 6 (Supplemental Figure S5, A and B). Leaf width was found to be strongly linearly correlated with leaf water potential (Supplemental Figure S1), meaning that leaf width could be used to indicate dynamic changes in leaf water potential. The transition to the rapid decline of *F_v_*/*F_m_* and leaf width occurred at 1,793.00 ± 13.70 and 2,083.70 ± 337.77 min of drought, respectively (Supplemental Figure S5). Stem water potentials of all plants behaved similarly, with Ψ_stem_ declining linearly from initial values of approximately −0.5 to −6 MPa over 5 days. Fluorescence and leaf width also showed a similar dynamic according to changes in Ψ_stem_, whereby initial static values (in the case of *F_v_*/*F_m_*) or a slow decline (in the case of leaf width) transitioned to a much steeper decline as water potential fell below a critical point (Figure 2, A and B). This change in slope occurred at similar values of Ψ_stem_ for *F_v_*/*F_m_* and leaf width (−3.53 ± 0.07 and −3.62 ± 0.43 MPa, respectively).

### Vulnerability to cavitation of leaf veins

**Figure 3:**
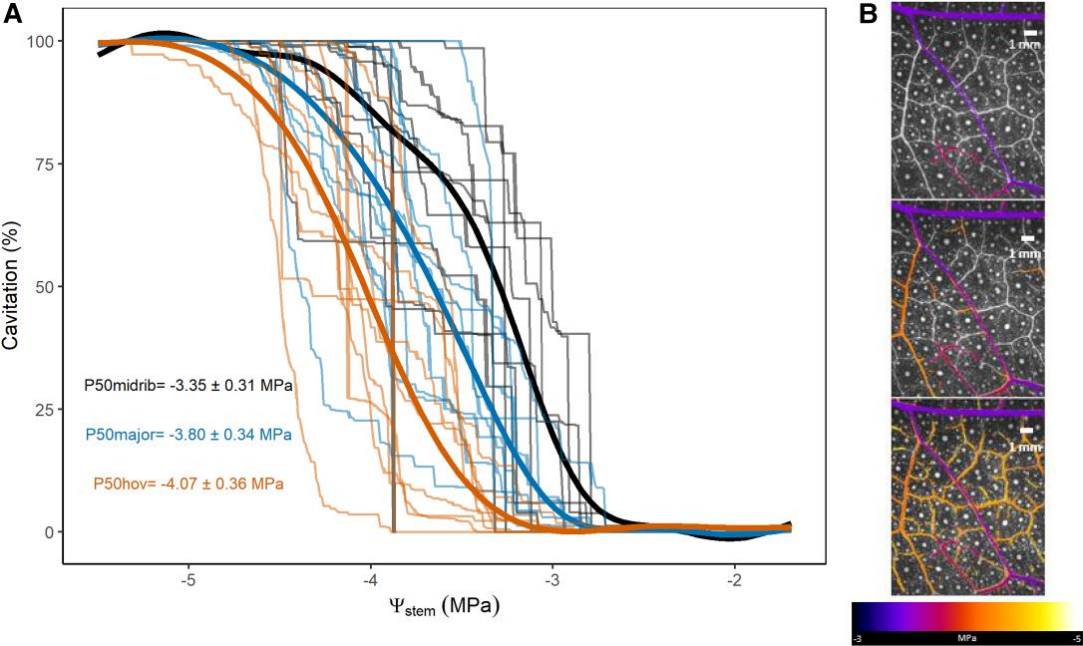
Cavitation spread in the different leaf vein orders. A, Relationships between stem water potential (Ψ_stem_) and cumulative cavitation in the midrib, major veins, and HOV of *E. viminalis* leaves (*n* = 13). The midrib is indicated in black, major veins in blue, and HOV in orange. B, Progression of cavitation spread at three different water potentials during the dehydration of a single plant. Cavitation events occurred between −3.0 and −5.0 MPa and are color coded according to the water potential at which they occurred (see scale). Midrib and major veins cavitated on average before HOV.

**Figure 4:**
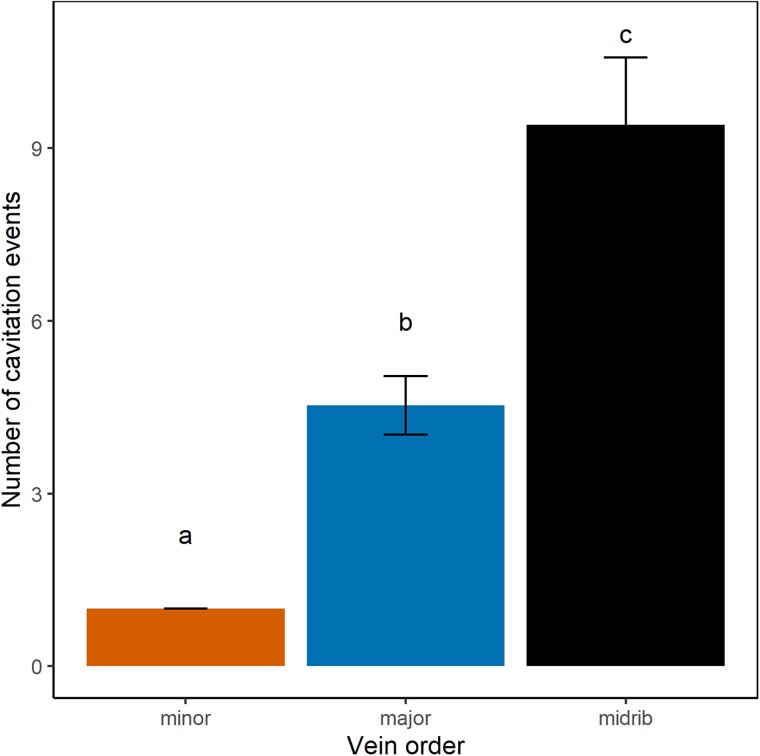
Mean number of cavitation events ± Sd according to the different vein orders (hov stands for high-order veins) in all leaves (*n* = 13). Different letters indicate significant differences between orders according to the ANOVA (*P*-value < 0.05).

Cavitation of the leaf xylem was investigated to determine if this provided an explanation for the acceleration in drying speed occurring coincidently with the onset of leaf damage. The mean stem water potential at which 50% of the leaf vein xylem was cavitated (P50_leaf_) ranged from −3.41 to −4.06 MPa (mean P50_leaf_ = −3.73 ± 0.33; *n* = 13; Table 1). However, a clear pattern was evident within leaves whereby cavitation initiated in the midrib and then spread to higher vein orders. When analyzed separately, the three vein orders displayed different temporal patterns of cavitation spreading (Figure 3A). On average the midrib was most vulnerable (mean P50_mid_ = −3.35 ± 0.38 MPa), followed by the major veins (mean P50_maj_ −3.80 ± 0.35 MPa) and HOV (mean P50_hov_ −4.07 ± 0.39 MPa; Figure 3A). Midrib vulnerability was also statistically different from that of major and HOV (*P*-value < 0.05; Supplemental Figure S6). The number of cavitation events also varied among vein orders (Figure 4). Midrib and major veins cavitated on average 9.4 ± 1.17 and 4.53 ± 0.51 times per vein segment, respectively. HOV cavitated progressively by groups of different sizes, but each segment only cavitated once.

**Table 1:** List of traits measured on *E. viminalis*

Abbreviation	Full name	Unit of measure	Mean ± Sd
Time *F*_v_/*F*_m_	Time corresponding to the breakpoint of fluorescence decline	min	1,793.00 ± 13.70
BP *F*_v_/*F*_m_	Water potential corresponding to the breakpoint of fluorescence decline	MPa	−3.53 ± 0.07
Time leaf width	Time corresponding to the breakpoint of leaf width decline	min	2,083.70 ± 337.77
BP leaf width	Water potential corresponding to the breakpoint on leaf width	MPa	−3.62 ± 0.43
TLP	Water potential at turgor loss point	MPa	−2.01 ± 0.01
P20_hov_	Water potential at 20% loss of high-order veins conduits	MPa	−3.79 ± 0.39
P50_leaf_	Water potential inducing 50% loss of leaf water transport capacity	MPa	−3.73 ± 0.33
P50_mid_	Water potential at 50% loss of midrib conduits	MPa	−3.35 ± 0.38
P50_maj_	Water transport capacity at 50% loss of major veins conduits	MPa	−3.80 ± 0.35
P50_hov_	Water potential at 50% loss of minor vein conduits	MPa	−4.07 ± 0.39
P50_stem_	Water potential at 50% loss of stem conduits	MPa	−3.97 ± 0.64
P88_stem_	Water potential at 88% loss of stem conduits	MPa	−5.45 ± 0.78
VPD	Vapor-pressure difference	kPa	1.94 ± 0.06
*g*_min_	Minimum cuticular conductance	mmol m^−2^s^−1^	10.02 ± 2.30
*E*_c_	Cuticular transpiration	mmol m^−2^s^−1^	0.19 ± 0.04
*K*_max_	Maximum hydraulic conductance	mmol s^−1^ m^−2^ MPa^−1^	5.77 ± 0.10
Runaway	Water potential corresponding to the point of hydraulic failure according to the Runway hypothesis	MPa	−3.82 ± 0.22
PLC runaway	Percentage loss of leaf hydraulic conductance	%	79.21

Mean values and standard deviations are reported.

**Figure 5:**
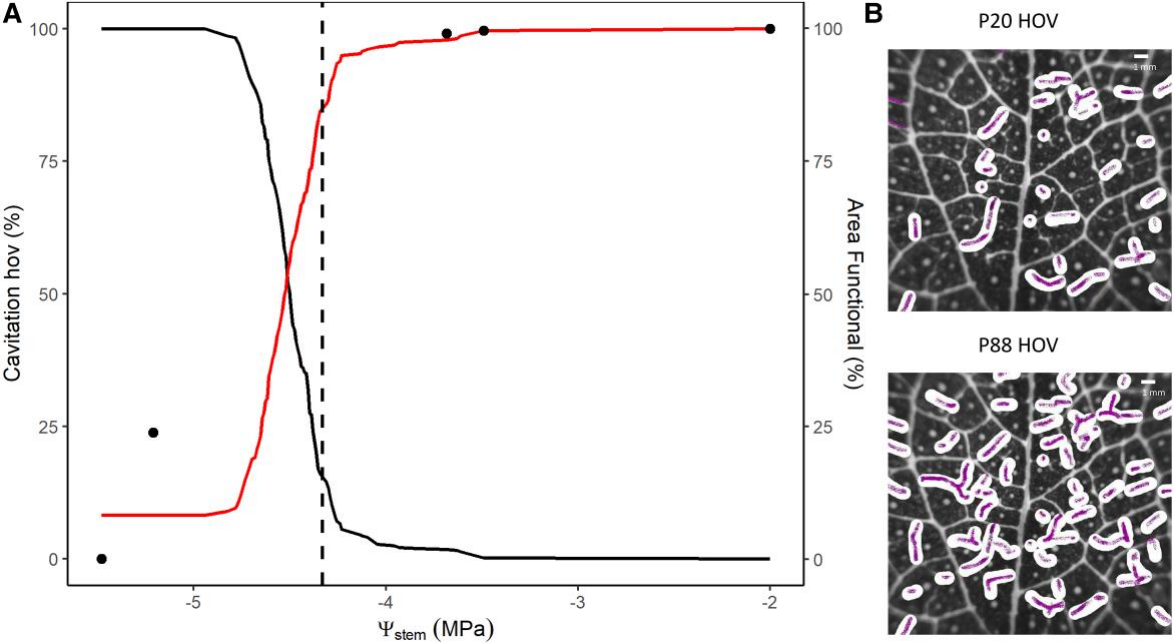
Loss of leaf functional area according to the spread of cavitation in HOV. A, Plot of HOV cavitation (black line) and leaf area remaining with fully functional HOV water delivery (% of total leaf area; red line) during dehydration in a specific single leaf of *E. viminalis*. The dashed black line represents the breakpoint in leaf width (see Figure 2B), black dots represent the decline of *F*_v_/*F*_m_ for this particular individual. B, White shading shows the amount of functional area loss at P20_hov_ (20% loss of HOV transport capacity) and P88_hov_ (88% loss of HOV transport capacity) for this particular plant (same sample as shown in Figure 1A).

Because HOV (veins from third order and above) were apparently deactivated by single cavitation events, thus completely severing xylem water supply to the downstream region of leaf tissue (Figure 4), the total leaf area supplied by the HOV declined in parallel with the increasing % of cavitation in this order (Figure 5).

Stems were less vulnerable to cavitation than leaves with a mean P50 of −3.97 ± 0.64 MPa, suggesting a degree of segmentation in *E. viminalis*. Due to the composite nature of the stem, there is still considerable undamaged xylem at −4.80 MPa, when HOV completely lose their functionality (P88stem = −5.45 ± 0.78, Table 1).

### Events during dehydration

**Figure 6:**
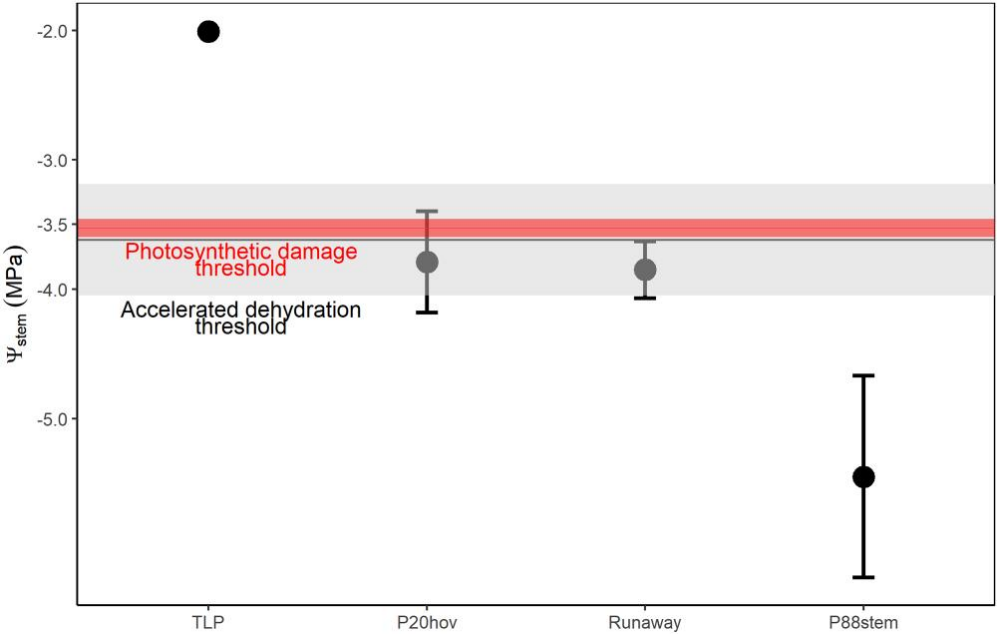
Water potential thresholds for events that may trigger leaf death during dehydration. The red horizontal line represents the average Ψ_stem_ (stem water potential) at which the breakpoint for *F*_v_/*F*_m_ (threshold for photosynthetic damage) was calculated; red area indicates standard deviation. The gray horizontal line indicates the average Ψ_stem_ at which the breakpoint for leaf width dynamic was calculated (threshold for accelerated dehydration); gray area represents standard deviation. Black symbols show median values ± Sd of different water potential thresholds: TLP; P20_hov_ (20% loss of HOV transport capacity); runaway (predicted point of runaway leaf cavitation), and P88_stem_ (88% loss of stem water transport capacity).

Based on a mean maximum *K*_leaf_ of 5.77 ± 0.10 mmol s^−1^ m^−2^ MPa^−1^, P50_leaf_ of −3.73 ± 0.33 MPa (Table 1 and Supplemental Figure S2), and *g*_min_ of 10.02 ± 2.30 mmol m^−2^ s^−1^, it was possible to calculate the theoretical water potential causing runaway cavitation in the leaf from (Eq. 2). Under average laboratory conditions of temperature (20°) and vapor-pressure difference (VPD, 1.94 ± 0.06 kPa), the runaway cavitation point would be −3.82 ± 0.22 MPa (Table 1 and Figure 6). The percentage loss of hydraulic conductance (PLC) associated with the runaway cavitation was 79.21% (Table 1).

Four processes were considered as mechanistic candidates for the rapid transition to tissue damage in leaves. The turgor loss point (potentially causing direct cell damage), incipient cavitation in HOV (due to their complete deactivation during cavitation), runaway cavitation threshold (causing leaf hydraulic failure), and high percentage of stem cavitation. The Ψ_stem_ at incipient leaf tissue damage (the *F*_v_/*F*_m_ inflection point) during drying overlapped with the values of Ψ_stem_ at which leaf shrinkage accelerated, and 20% of HOV area was cavitated (P20_hov_ = −3.79 ± 0.39 MPa) (Figure 6). In terms of the timing of these events, we also found a strong relationship between the breakpoint in leaf width and 20% HOV cavitation, suggesting a tight connection in time and water potential (Supplemental Figure S7, A and B). Over an average drying time of 2,116.15 ± 542.85 min, there was an average of 17.92 ± 88.66 min time difference between 20% HOV and breakpoint (BP) shrinkage. Other vein orders had larger residuals (Supplemental Figure S8). Comparing Ψ_stem_ at BP leaf width also showed a very small mean residual between 20% HOV and tissue accelerated dehydration (0.01 ± 0.13 MPa) compared with larger values for other vein orders (Supplemental Figure S8).

Mean water potentials at P88_stem_ and turgor loss point (TLP) were substantially different to the slope BP in *F*_v_/*F*_m_. The point of runaway cavitation was, however, not different from the water potential at the onset of a rapid decline in leaf width and *F*_v_/*F*_m_, suggesting a mechanistic link.

## Discussion

During dehydration of whole *E. viminalis* plants we identified runaway cavitation and incipient HOV cavitation as a critical threshold beyond which leaves became quickly damaged. Simultaneous measures of leaf shrinkage, leaf photosynthetic damage, and xylem cavitation (Figure 6) demonstrated how minutes after the spread of cavitation into the terminal part of leaf vasculature leaf dehydration greatly accelerates triggering the loss of mesophyll cell photosynthetic function.

### Indicators of incipient leaf tissue damage

We found clear evidence that leaf tissue damage, quantified by the decline in photosystem II fluorescence (*F*_v_/*F*_m_; Supplemental Figure S5A and Figure 2A), occurred once leaf water potential reached a critical level, as has been reported in studies on diverse species (Zia et al., 2016; Cardoso et al., 2018; Johnson et al., 2018; Trueba et al., 2019; Brodribb et al., 2021). We also detected a strong association between leaf shrinkage and leaf damage, suggesting that the proximate cause of cell damage during dehydration is likely to be membrane damage (Mittler, 2002; John et al., 2018; Johnson et al., 2018; Trueba et al., 2019; Brodribb et al., 2021).

If it is assumed that damage occurs at a critical stage of leaf shrinkage, then the strong association between tissue hydration (water potential) and tissue width seen here (Supplemental Figure S1) and in other studies (Lamacque et al., 2020; Skelton, 2020; Bourbia et al., 2021) provides an excellent tool for monitoring the dynamic process of leaf dehydration and damage. Of particular importance is the evidence here of a clear breakpoint in leaf width during the dehydration process where the rate of leaf dehydration substantially increased (Supplemental Figure S5B and Figure 2B). Indeed, measures of leaf dehydration, like shrinkage and relative water content can predict desiccation thresholds and mortality risk (Blackman et al., 2019; Martinez-Vilalta et al., 2019; Sapes et al., 2019; Mantova et al., 2022).

In our case, the increase in cellular dehydration (close to the point of lethal desiccation) corresponded to xylem cavitation in the terminal part of the leaf vein network, providing a likely causal association. The concurrency of breakpoints in relationships between water potential and both leaf width and photosystem damage suggested a sequence of events whereby mesophyll cell damage is triggered by increased mechanical stress brought on by an acceleration in the rate of leaf dehydration after leaf tissue is disconnected from the xylem following vein cavitation. A similar sequence of events was recently observed in tomato (*Solanum lycopersicum*) leaves exposed to extreme transpiration stress (Brodribb et al., 2021), but in this previous case, the rapid spread of cavitation and damage (within seconds of the initial cavitation) prevented any conclusions as to the structure of vein network cavitation responsible for lethal dehydration. In contrast, here in a woody tree species subjected to much slower dehydration, we resolved the critical importance of cavitation spread in the vein network as a trigger point for leaf damage.

### Vulnerability to cavitation and redundancy of leaf veins

Consistently with previous authors (Brodribb et al., 2016; Scoffoni et al., 2017; Cardoso et al., 2020; Petruzzellis et al., 2020), we observed a temporal variation in the initiation of embolism spread in different leaf vein orders (Figure 3, A and B). Importantly, we also found large differences in the total number of cavitation events that occurred in discrete lengths of the different vein orders (Figure 4), indicating a large range in vessel redundancy within veins (the number of functional vessels remaining after cavitation events) and between vein orders. These results highlight the substantial redundancy in the leaf network during the early spread of cavitation through the midrib and major veins which enables a continuous central water supply despite serious damage by cavitation (Mauri et al., 2020). Consequently, even if major and HOV had similar vulnerability (Figure 3A and Supplemental Figure S6), the redundancy in pathways for water flow in major veins means that it is unlikely that the early stages of cavitation in the larger veins would disrupt water delivery sufficiently to cause leaf dehydration and damage. Even after 50% of larger veins are cavitated, the water transport capacity to the mesophyll provided by the large number of xylem conduits that remain functional would be sufficient to support the very low transpiration rate in water-stressed leaves (Plymale and Wylie, 1944). In contrast, our data show segments of HOV to be completely blocked by a single cavitation event. We interpret the fact that all HOV behaved similarly to indicate that cavitation spread to all radially neighboring vessels in these small veins, deactivating them in a single recorded event. A consequence of nonredundancy in HOV during water-stress-induced cavitation is that the local water supply to small regions of the leaf became progressively severed, leading to rapid desiccation and damage to downstream regions of photosynthetic tissue. We observed that the 20% of HOV cavitation (corresponding to a 20% of vein length blockage) matched the water potential inducing photosynthetic damage and acceleration in the dehydration process (Figure 6 and Supplemental Figure S7). In particular, synchronicity in the timing of incipient HOV cavitation and the breakpoint of accelerated dehydration (Supplemental Figure S7), strongly suggests causality. Comparisons with other vein orders, or stem cavitation showed weaker time associations with shrinkage and tissue damage (Supplemental Figure S8).

Alternatively, it has been hypothesized that declining photosynthetic capacity during drought is triggered by midrib cavitation (Nardini and Salleo, 2003; Sack et al., 2003; Cardoso et al., 2018). This was not the case in *E. viminalis* where midrib cavitation occurred at water potentials substantially less negative than the incipient point of leaf damage.

Previous studies have found strong associations between minor vein anatomy and drought tolerance, showing that structural reinforcement of HOV (generally referred to as minor veins) is strongly associated with hydraulic vulnerability (Blackman et al., 2010) verifying their importance in setting water-stress limits (Nardini et al., 2012; Kawai and Okada, 2018). In addition, the density of leaf HOV plays a major role in determining the efficiency of leaf water transport and maximum rates of photosynthesis (Brodribb et al., 2007; Sack and Scoffoni, 2013). The likelihood that HOV cavitation triggers leaf damage adds another feature to the central role of this terminal part of the water transport pathway.

Our data demonstrate a clear sequence linking runaway cavitation in the leaf veins with rapid shrinkage and mesophyll damage. HOV cavitation appears to be the terminal phase of vascular disconnection and further work in other species will be required to determine whether this pattern proves to be universal.

The close proximity between the water potential calculated to trigger runaway cavitation in leaves and the water potential measured at 20% HOV cavitation suggests these events may be interconnected. Due to the nonredundancy of HOV cavitation, any level of cavitation in this order would be predicted to lead to a dramatic reduction of water supply to downstream tissue and the severing of local water supply. We hypothesized that this would not only lead to lethal local dehydration, but also to the propagation of cavitation into neighboring parts of the xylem network, leading to a type of runaway cavitation. We were not able to differentiate these two mechanisms that are in fact providing an equally viable explanation for the lethal acceleration of leaf dehydration, and we postulate that this combination of effects drives rapid leaf death.

Interestingly, we also noticed that the level of leaf embolism associated with runaway cavitation was not as high as we would have expected. Runaway cavitation has been traditionally theorized to occur close to 100% embolism (Tyree and Sperry, 1988) and a recent work by Johnson et al. (2022) suggested runaway cavitation spread in branchlets of Oyster bay pine (*Callitris rhomboidea*) at >90% stem embolism. Using a derivation of the runaway cavitation point, here we found that the runaway point in *E. viminalis* was 79.21% PLC (Table 1). This explicit derivation of the runaway point using hydraulic theory presents opportunities to explore the impact of key components of the runaway process, such as the shape of the xylem vulnerability curve, and the role of cuticular conductance.

HOV vulnerability appears to provide a more mechanistically robust metric to quantify the drought resistance of species than other metrics, such as TLP, P88_stem_ or P50_leaf_, especially for understanding canopy desiccation. However, in the case of *E. viminalis* presented here, these P88_stem_ and P50_leaf_ metrics corresponded closely with our thresholds of damage, possibly reflecting the minimal segmentation found in this species. We speculate that this might not be a general case in different species with diverse leaf habits and much stronger segmentation (Tyree et al., 1993; Pivovaroff et al., 2014; Rodriguez-Dominguez et al., 2018).

### Conclusion

We aimed to understand the role of catastrophic failure in the leaf supply network (either by runaway xylem cavitation and/or local failure of HOV) in triggering dehydration-induced damage in water-stressed leaves in a common woody tree species. The timing of incipient leaf death was pinpointed as a transition from an initial phase of controlled dehydration to a phase of accelerated tissue desiccation and photosynthetic damage. This transition point corresponded with the observation of incipient cavitation in the nonredundant minor vein network, providing a logical explanation for lethal tissue desiccation in *E. viminalis*. HOV cavitation also coincided with the predicted point of runaway leaf cavitation which was analytically derived. Although traditionally the plant lethal water potential is often associated with a high percentage of stem cavitation (50%–88%) loss of xylem function (Brodribb and Cochard, 2009; Urli et al., 2013), we argue that in *E. viminalis* it is not a percentage of stem water transport capacity loss that determines leaf death but rather the spread of cavitation into the final order of leaf veins where it triggers rapid and lethal local tissue desiccation. Further research will determine whether this is a more general pattern in trees, or whether, like in grasses (Johnson et al., 2018) tissue death can be triggered before the point of HOV cavitation.

## Materials and methods

### Plant material

Manna gum (*E. viminalis*) seeds were sampled in late 2019 from open-pollinated families located in a common garden at the University of Tasmania. Seeds were germinated and grown in 3L pots filled with potting mix consisting of an 8:4 mixture of composted pine bark and coarse washed river sand, in a glasshouse facility at the University of Tasmania from January to February 2020. Plants were watered daily and experienced natural light conditions and an average day and night temperature of 23°C and 18°C, respectively. As eucalypts have been reported to be susceptible to high temperatures and humidity typically found in the glasshouse (Ammitzboll et al., 2018), after 2 months the plants were moved outside into a common garden at the university, where they were watered twice daily. Physiological measurements were taken once the eucalypts had reached 6 months of age (approximately 140 cm tall), from April to July 2020.

### Plant dehydration

To determine the sequence and timing of events during plant dehydration, water was withheld from six-month-old *E. viminalis* saplings (*n* = 13) while simultaneously monitoring changes in leaf width, xylem cavitation, photosynthetic activity, and leaf and stem water potential (see details below). Before dehydration, the roots of fully hydrated *E. viminalis* saplings were gently rinsed in water to remove all the soil. This ensured that dehydration to the point of leaf death occurred within approximately 5–6 days. Plants were then transferred to the laboratory where the average temperature and relative humidity were 23°C and 35%, respectively. Psychrometers and cameras (optical vulnerability technique, OVT) were fitted to each plant to monitor water potential and cavitation (see details below). Plants were then allowed to dehydrate until the leaves became visibly damaged (i.e. discolored and desiccated).

### Water potential measurements

Stem (Ψ_stem_) and leaf water potential (Ψ_leaf_) were monitored continuously during plant dehydration. Prepared plants that were fully hydrated and free of soil were first enclosed in plastic bags with moist paper for 30 min. Halting transpiration in this way was assumed to cause the water potential of different plant organs to come into equilibrium. We recorded the plant water potential with psychrometers (ICT PSY Armidale, NSW, Australia) attached to the leaf and the stem of each plant (i.e. two psychrometers per plant). The stem psychrometer was attached about 10 cm above the root collar. To do this, the bark was carefully removed with a razor blade, and the xylem surface was gently rinsed with distilled water before drying with a tissue to remove any particles that would affect the water potential readings. The psychrometer was sealed to the stem tissue with parafilm to prevent moisture loss. Another psychrometer was attached to a leaf adjacent to that used for cavitation monitoring. A small region of the leaf surface was gently abraded with 1,000 grit sandpaper, positioned under the psychrometer chamber and the interface between leaf tissue and the sensor was sealed with a thin layer of high-vacuum silicone grease (Dow Corning, Midland, MI, USA). Leaf water potential was also measured after equilibration every 2 h during the day with a Scholander pressure chamber (PMS, Albany, OR, USA) for cross validation. As in Guan et al. (2021), we found good agreement between stem and leaf water potential measured with psychrometers until samples were substantially dehydrated (e.g. approximately −5 to −6 MPa). At this point, water potential decreased faster in the leaf than in the stem.

Lastly, we measured leaf shrinkage as a proxy for leaf water potential due to the likelihood of disequilibrium in water potential between leaves and the stem at very high levels of cavitation. This provided a means of monitoring water potential in the same leaf where cavitation was being recorded using the OVT (see below). To do this, we used the same images taken using the OVT (Brodribb et al., 2016) to measure the reduction in leaf width (shrinkage) during dehydration. Shrinkage was measured using IMAGEJ (National Institutes of Health, Bethesda, MD, USA) as the distance between the edge of the midrib and the leaf margin, parallel to major veins. To test the shape of the relationship between leaf shrinkage and water potential, 14 leaves from 5 fully hydrated plants were monitored during dehydration on a commercial flatbed scanner (Perfection V800, Epson America). Fully hydrated leaves were excised from the plant and quickly secured onto the scanner bed by carefully taping the tip and the base to prevent movement while still allowing shrinkage. The first scan provided a reference value for full hydration, after which scans were made every 5 min and leaves removed at different times to determine the water potential. Leaf water potential was measured with a psychrometer using leaf discs taken after the samples were scanned. We found a strong positive linear correlation between leaf width, expressed as a percentage of the maximum width at full hydration (*y* = 6.1963*x* + 106.22; *P*-value < 0.05) with leaf water potential (Supplemental Figure 1). There was no change in the slope of this relationship between −1.8 MPa (before TLP) and −4.4 MPa (threshold for leaf high % of embolism). On this basis, we used leaf width to infer whether changes in leaf width could be attributed to leaf water status.

### Turgor loss point

We measured the water potential at leaf turgor loss to determine if this had any effect on the onset of leaf damage. Five leaves were collected from five fully hydrated plants (one from each plant) and enclosed in a plastic bag for 30 min to allow the water potential of different tissues within the leaf to equilibrate. The water potential corresponding to the TLP was measured by pairing measurements of water potential using the pressure chamber and water loss was measured with a balance (± 0.0001 g, model MS204S; Mettler Toledo) during dehydration and by analyzing the resulting pressure–volume (PV) curve (Tyree and Hammel, 1972). Leaf water potential and weight loss were measured until leaves reached approximately −4.5 MPa at which point damage was evident. As juvenile leaves of *E. viminalis* are sessile (i.e. lack a petiole), PV curves were measured on terminal shoots bearing three to four mature leaves of the same age and size. As this part of the plant was small (<20 mm in length and 3 mm in stem diameter) we assumed that the parameters calculated would be largely determined by the leaf tissue characteristics.

### Leaf and stem hydraulic vulnerability to cavitation

To determine leaf xylem vulnerability to water-stress-induced cavitation, we analyzed the spread of cavitation through the veins with the OVT (Brodribb et al., 2016) while leaf water potential (Ψ_leaf_) and stem water potential (Ψ_stem_) were monitored during dehydration (as above). The OVT quantifies xylem cavitation by detecting changes in light reflection as water-filled vessels become air-filled, producing a temporally and spatially resolved map of cavitation in situ during dehydration. The technique has been verified in multiple studies showing strong correspondence with hydraulic and x-ray tomographic methods (Gauthey et al., 2020; Johnson et al., 2020; Cardoso et al., 2022) as well as pneumatic methods (Guan et al., 2021).

To capture the temporal and spatial pattern of cavitation spread in the leaf, we used multiple custom-built cameras (Cavicam, Hobart, Tasmania). Cameras were attached to the distal quarter of the leaf, covering an area of approximately 1 cm^2^, to view the midrib and the higher vein orders (Figure 1). Images were captured during dehydration every 5 min for approximately 5 days until no new cavitation events were recorded for 12 h. Image sequences were then analyzed to quantify differences in light transmission caused by xylem cavitation using image subtraction in IMAGEJ (for details, see Brodribb et al., 2016 and www.opensourceov.org). Combining data from the dynamics of declining water potential with cavitation data for each leaf enabled the accumulation of cavitation events to be plotted as a function of Ψ_stem_. A cumulative projection of all the threshold images produced a map of all embolism events and permitted cavitation in different vein orders (midrib, major veins, and HOVs) to be individually identified. To test the possibility that incipient minor vein cavitation was responsible for tissue death, we quantified the Ψ_stem_ causing 20% cavitation in HOV (P20_hov_) as a likely trigger of leaf damage as quantified by chlorophyll fluorescence (see below).

We identified three vein categories; the midrib being the central vein departing from the leaf insertion point (juvenile leaves of *E. viminalis* are sessile); major veins as those veins that departed from the midrib; HOV were considered all the smaller veins (third order and above, and diameter smaller than 50 μm; Figure 1, A and B).

We also determined the progressive loss of leaf area supplied by xylem during the cavitation of HOV in a single representative leaf with average cavitation vulnerability. A total of approximately 70 HOV segments were measured for this calculation. The unique area supplied by each HOV was assumed to be half the distance to the nearest neighboring vein (Brodribb et al., 2007). Based on the OVT image sequence where only HOV were considered, we used the paintbrush tool in IMAGEJ set to a diameter equal to the average distance between neighboring HOV to highlight the tissue affected by every minor vein cavitation event. We then plotted the area against Ψ_leaf_.

Stem vulnerability to xylem cavitation was also measured in a subset of six plants using the OVT as in Brodribb et al. (2017) to compare the vulnerability of leaves and stems. To do this a window of bark was carefully removed from young stem segments, close to the plant apex to view the xylem. An adhesive hydrogel (Tensive Conductive Adhesive Gel, Parker Laboratories) was applied to the exposed xylem to prevent water loss. The custom-built camera setup described above was installed on the stem with reflected light used to detect cavitation events rather than transmitted light.

### Leaf damage

Leaf damage was monitored during dehydration using chlorophyll fluorescence as a measure of photosynthetic impairment. The optimum quantum yield of photosystem II was measured in the middle of the leaf by applying a saturating pulse of light and measuring the ratio of maximum to variable fluorescence (*F*_v_/*F*_m_). We measured the change in *F*_v_/*F*_m_ on a subsample of six plants chosen among those monitored for dehydration and cavitation. Eight to ten leaves per plant (neighboring the leaf monitored for cavitation, as *E. viminalis* has opposite leaves) were monitored for *F*_v_/*F*_m_ during dehydration. We alternated measures of fluorescence among leaves wrapped in aluminum foil for 2 h to dark adapt the tissue before using a portable fluorometer (PAM-2000, Walz GmbH, Effeltrich, Germany, diameter of fluorescence beam approximately 20 mm). Leaves were measured every 3 h from dusk to dawn and once the light was switched off until the point of leaf death (fluorescence of photosystem II *F*_v_/*F*_m_ < 0.2). Three control plants were used to verify that the decline in fluorescence was specifically associated with the dehydration treatment and not to senescence or laboratory conditions. Plants were kept well watered, and *F*_v_/*F*_m_ measurements were taken daily over 5 days (which was the same amount of time allocated in the dehydration experiment). We did not find any variation in *F*_v_/*F*_m_ (Supplemental Figure S2).

### Runaway cavitation

We formulated the theoretical derivation of the point of runaway leaf cavitation based on parameters of leaf hydraulic conductance (*K*_max_), the water potential at 50% loss of *K*_max_ in the leaf (P50), the slope of the vulnerability curve (*α*), and the cuticular transpiration (*E*_c_).

The vulnerability of *K* to declining water potential was approximated as a sigmoidal curve described as:


(1) $$K=\frac{K_{\max}}{1+e^{(\Psi -P50)/\alpha }}$$

where *K* is the hydraulic conductance (mmol m^−2^ s^−1^ MPa^−1^), *K*_max_ is the maximum hydraulic conductance (mmol m^−2^ s^−1^ MPa^−1^), Ψ is the bulk leaf water potential (MPa), P50 is the water potential at which 50% of hydraulic conductance is lost (MPa), and *α* is the slope of the vulnerability curve (MPa).

The water potential at which runaway cavitation occurs was calculated as:


(2) $$\Psi =P50-\alpha \ln\;\left(\frac{\alpha K_{\max}}{E_{c}}\right)$$

We also calculated the PLC (percentage loss of leaf hydraulic conductance at runaway cavitation) as:


(3) $$\mathrm{PLC}=\left(\frac{\frac{\alpha K_{\max}}{E_{c}}}{1+\frac{\alpha K_{\max}}{E_{c}}}\right)\times 100\;.$$

where *E*_c_ is the cuticular transpiration (mmol m^−2^ s^−1^) and was calculated as:


$$E_{c}=\;g_{\min}\times \frac{\mathrm{VPD}}{P_{\mathrm{atm}}}$$

where *g*_min_ is the minimum cuticular conductance (mmol m^−2^ s^−1^), VPD is the vapor-pressure deficit, and *P*_atm_ is the atmospheric pressure (kPa). Temperature and humidity conditions at which these measurements were taken were, respectively, 24.31 ± 0.95°C 35.86 ± 5.67%. The derivations for Equations 1–3 are available in the Supplemental Material (Method S1).

### *K*_leaf_ and *g*_min_ measurements

Calculating the theoretical runaway cavitation point using (Eq. 2) required knowledge to fit a sigmoidal function to the leaf hydraulic conductance (*K*_leaf_) vulnerability curve. As the optical method does not yield such a function, we used the kinetic rehydration technique (Brodribb and Cochard, 2009) to calculate *K*_leaf_ at different water potentials. *K*_leaf_ measurements were performed in February 2021–2022 on four field-grown individuals; these plants were germinated from the same seed pool we used for the dehydration experiment, grown in the same conditions and were of the same age. *K*_leaf_ was measured by quantifying the water flux into rehydrating leaves that had been dehydrated to a range of water potentials from −0.5 to −5.7 MPa. Branches collected from four well-hydrated plants were collected from a common garden trial and immediately transferred to the laboratory. The branches were enclosed in plastic bags for 30 min to enable the water potential of different tissues to equilibrate before they were allowed to dehydrate. Leaf water potential was measured periodically using a Scholander pressure chamber and measurements were performed once a range of different water potentials was reached. Shoots (around 20 cm in length and approximately 2 mm in diameter) were collected from samples at different water potentials and recut underwater (to remove any embolized xylem that might originate from the initial cut) and immediately attached to a microflowmeter (Brodribb and Holbrook, 2006) filled with filtered degassed water at room temperature (23°C) to monitor the flow rate of water into the leaf. Water flow was logged every 1 s until the maximum flow (*F*_max_) was reached. The branch continued to rehydrate until flow reduced to approximately half (*F*_2_) at which point the branch was disconnected, wrapped in a damp paper towel and after equilibrating for 30 min in a plastic bag, the final water potential (Ψ_fin_) was measured. Two instantaneous values of *K*_leaf_ were calculated from *K*_leaf_ = *F*_max_/Ψ_init_ and *K*_leaf_ = F_2_/Ψ_fin_ for each sample. The variation between the two values of *K*_leaf_ calculated per leaf sample was typically less than 10% enabling the average of the two values to be used as a mean measure of maximum hydraulic conductance which was normalized by leaf area and standardized to the viscosity of water at 20°C (see Supplemental Figure S3 for *K*_leaf_ curve).

The model also required cuticular transpiration (*E*_c_, mmol m^−2^ s^−1^), which was obtained from minimum cuticular conductance (*g*_min_, mmol m^−2^ s^−1^) by measuring five fully hydrated leaves from two individuals belonging to the same cohort of *E. viminalis* used in the dehydration experiment. Leaves were detached from plants, cut ends were sealed with silicone grease, and water loss was measured by weighing the leaves every minute using an electronic balance (± 0.0001 g, model MS204S; Mettler Toledo, Columbus, OH, USA), while the light intensity during the experiment was ca. 1000 μmol m^−2^ s^−1^. The temperature (24.31 ± 0.95°C) and relative humidity (35.86 ± 5.67%) during the experiment were recorded every minute with an HMP45AC temperature-humidity probe (Campbell Scientific Inc.) and CR850 datalogger (Campbell Scientific). Following Duursma et al. (2019), leaf weight was measured until a steady-state phase was reached, in which stomata closed and water loss became stable (after ca. 1 h, see Supplemental Figure S4). Once leaves reached this phase, they were rehydrated for 30 min and scanned to measure leaf area. *g*_min_ was calculated from the linear portion of the curve during this last phase as:


$$g_{\min}=\frac{\mathrm{WL}\times \;P_{\mathrm{atm}}}{\mathrm{VPD}}$$

where WL is the water loss rate (mmol m^−2^ s^−1^) obtained as the slope of mass (g) over time (s) and normalized by area (m^2^); *P*_atm_ is the atmospheric pressure (101.3 kPa) and VPD is the vapor-pressure deficit. VPD was calculated with the Arden–Buck equation (Buck, 1981), assuming that the internal airspaces in the leaf were saturated with water vapor:


$$\mathrm{VPD}=\left(1-\frac{\mathrm{RH}}{100}\right)\left(0.61121\times \;e^{\;\frac{17.502T}{240.97+\;T}}\right)$$

where RH is the relative humidity (%) and *T* is the air temperature (°C).

### Statistical analysis

Data were analyzed using R version 3.6.2 (R Core Team, 2016). To determine the point at which there was a rapid increase in leaf dehydration, we fitted a local polynomial regression (loess function) to the relationship between Ψ_stem_ and leaf width and then calculated the breakpoint (leaf water potential at a change in slope) with the function *segmented* in the package “segmented,” version 1.1 (Muggeo, 2008). In brief, this function guesses an initial breakpoint by iteratively fitting a standard linear model and updating the breakpoint (calculated by the two fitted straight lines) until the algorithm converges, thus also providing with a standard error. We calculated a breakpoint for each individual (*n* = 13) and used the same approach for the relationship between water potential and *F*_v_/*F*_m_ (*n* = 6).

Generalized additive models (GAMs) were fitted on vulnerability curves to extrapolate the mean values associated with different vein order cavitation.

Changes in *K*_leaf_ according to Ψ_leaf_ was determined using the regression fitting software Sigmaplot version 12.5 (Systat Software Inc., San Jose, CA, USA) to fit a four-parameter sigmoid function of the form *y* = *y*_0_ + *a*/(1 + exp(−(Ψ − *x*_0_)/*b*)).

Two-way parametric ANOVA analysis was used to test for the differences between the number of cavitation events according to vein order, and among different thresholds of cavitation with the *aov* function in the “stats” package in R software. After checking for data normality and homogeneity of variance, post hoc Tukey's honestly significant difference comparison was run with the *TukeyHSD* function in the “stats” package. The same approach was used to test differences between P50 in the different vein orders.

## Supplementary Material

kiad016_Supplementary_Data

## Supplemental data

The following materials are available in the online version of this article.

**Supplemental Figure S1**. The relationship between leaf water potential and leaf width (% of maximum) measured on 14 detached leaves of *E. viminalis* is described by a strong linear correlation.

**Supplemental Figure S2**. Measures of fluorescence (*F*_v_/*F*_m_) for three controls plants that were monitored for 5 days in the same laboratory condition used for the dehydration experiment.

**Supplemental Figure S3**. Plot of leaf hydraulic conductance (*K*_leaf_, mmol m^−2^ s^−1^ MPa^−1^) and leaf water potential (Ψ_leaf_, MPa).

**Supplemental Figure S4**. Example of plot of stomatal conductance (*g*_s_, mmol m^−2^ s^−1^) through time (s) for one leaves of *E. viminalis*.

**Supplemental Figure S5**. Changes to leaf fluorescence (*F*_v_/*F*_m_) (A, *n* = 6) and width (B, *n* = 13) during time (min).

**Supplemental Figure S6**. Leaf water potential inducing a 50% loss of water transport capacity (P50, MPa) in the midrib, major, and HOV of *E. viminalis* leaves (*n* = 13).

**Supplemental Figure S7**. The relationship between the breakpoint in leaf width and 20% HOV cavitation, suggesting a tight connection in time and water potential.

**Supplemental Method S1**. Point of runaway vulnerability with full sigmoidal curve.
