# Supplementary material for: Deadly acceleration in dehydration of Eucalyptus viminalis leaves coincides with high-order vein cavitation
Source: Plant Physiol. 2023 Jan 24;191(3):1648–61. doi: 10.1093/plphys/kiad016 (PMC10022613; doi:10.1093/plphys/kiad016)
Supplement: kiad016_Supplementary_Data [file kiad016_supplementary_data.pdf]

***Plant Physiology* Supporting Information**

Article title: Deadly acceleration in the dehydration of *Eucalyptus viminalis* leaves coincides with high order vein cavitation
Authors: Vanessa Tonet, Madeline Carins-Murphy, Ross Deans, Timothy Brodribb

The following Supporting Information is available for this article:

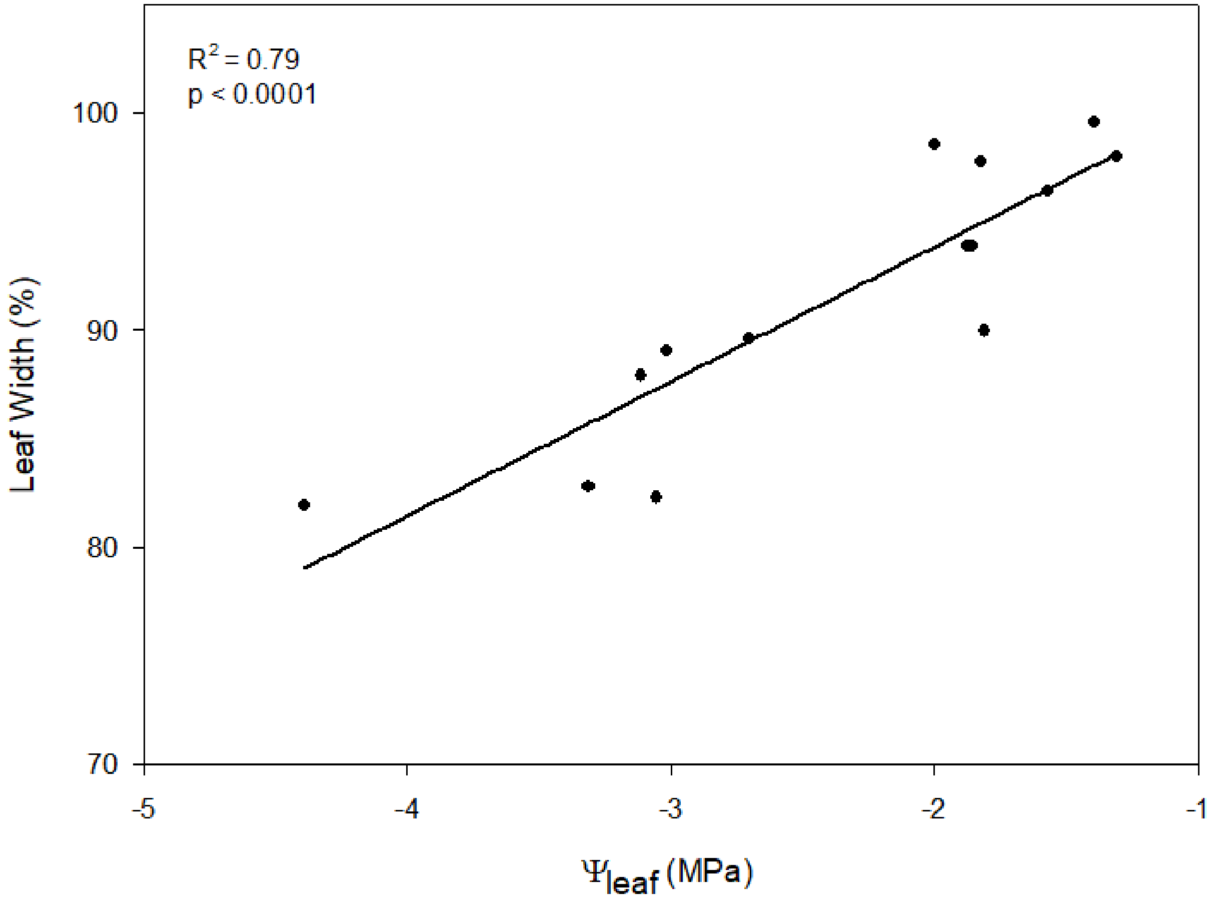

Supplemental Figure S1 The relationship between leaf water potential and leaf width (% of maximum) measured on 14 detached leaves of *E. viminalis* is described by a strong linear correlation.

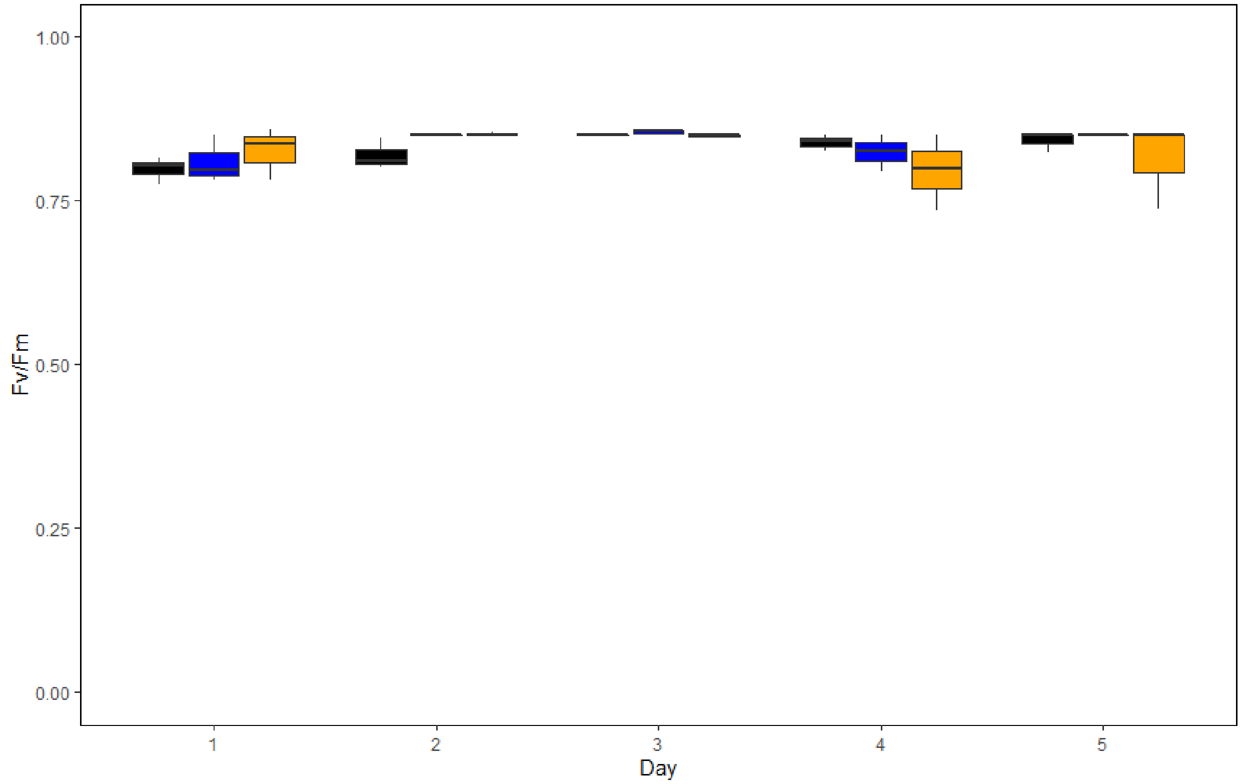

Supplemental Figure S2 Measures of fluorescence (Fv/Fm) for 3 controls plants that were monitored for 5 days in the same laboratory condition used for the dehydration experiment.

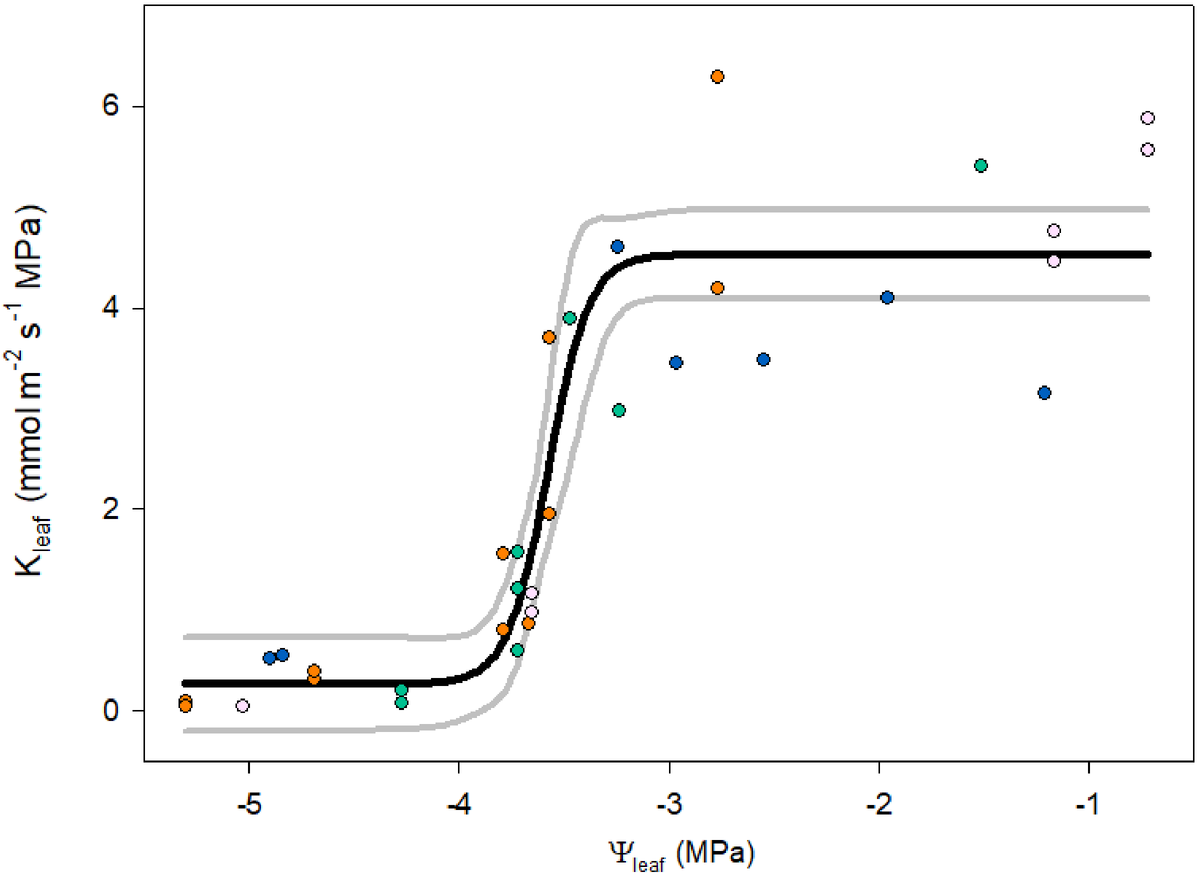

Supplemental Figure S3 Plot of leaf hydraulic conductance (K_{leaf}, mmol m^{-2} s^{-1} MPa^{-1}) and leaf water potential (Ψ_{leaf}, MPa). Different colors indicate different values of K_{leaf} from four individuals, black line is the K_{leaf} described by a sigmoidal function. Grey lines indicated 95% confidence interval.

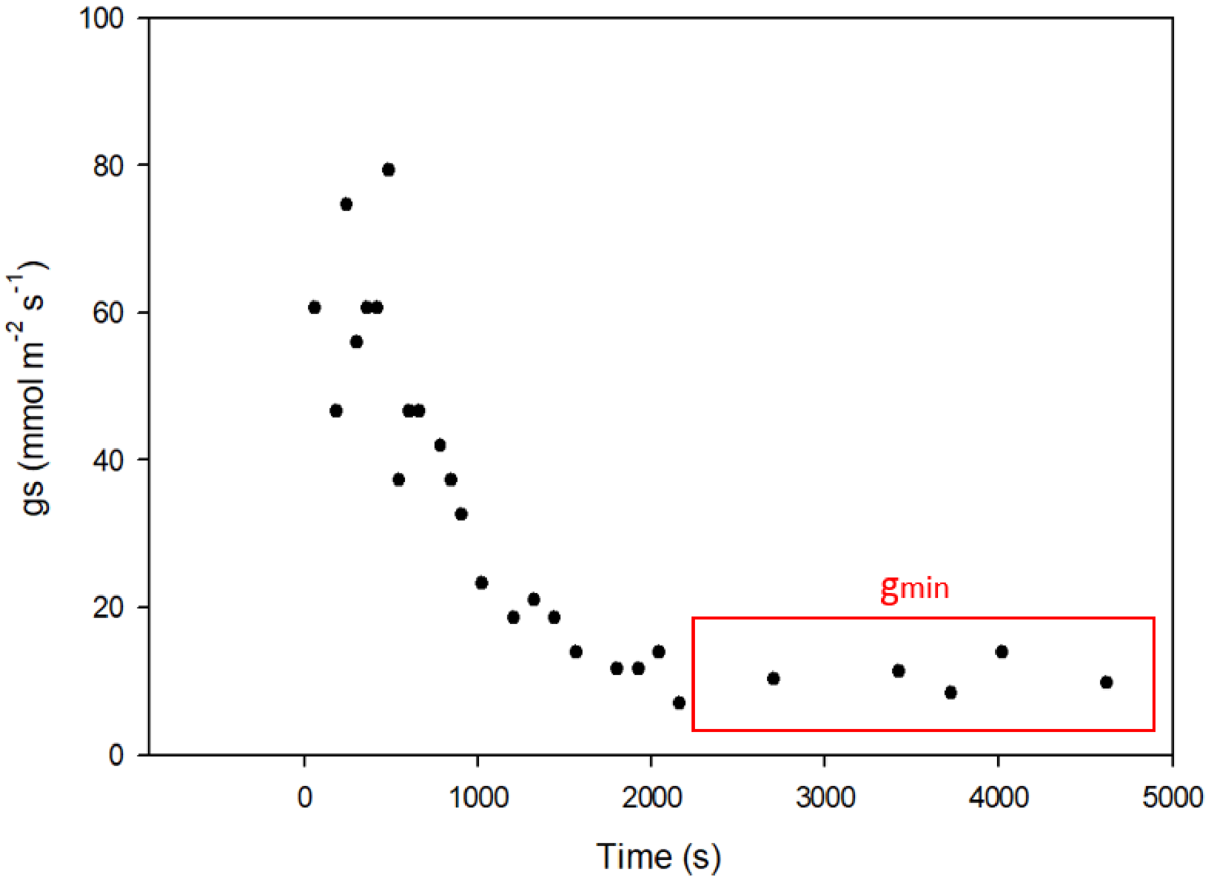

Supplemental Figure S4 Example of plot of stomatal conductance (gs, mmol m^{-2} s^{-1}) through time (s) for one leaves of *E. viminalis*. The red box shows the steady state phase (when stomata are closed) that was used to calculate gmin (mmol m^{-2} s^{-1}).

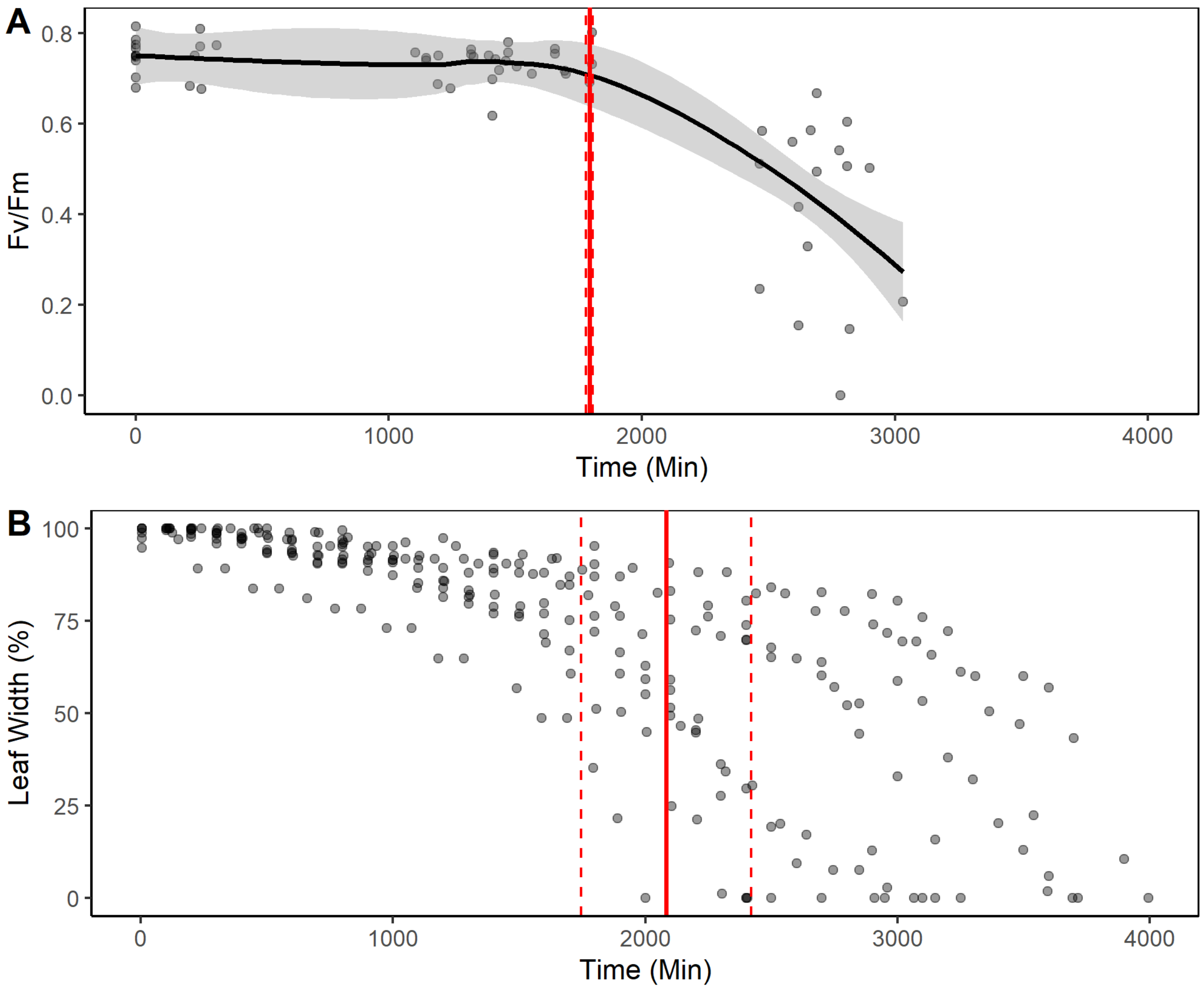

Supplemental Figure S5 Changes to leaf fluorescence (Fv/Fm) (A, n=6) and width (B, n=13) during time (Min). Solid black lines are regressions with 95% confidence intervals (light grey shading). Red solid lines represent the mean time at the slope breakpoint in Fv/Fm and leaf width respectively, and dashed lines represented the std associated.

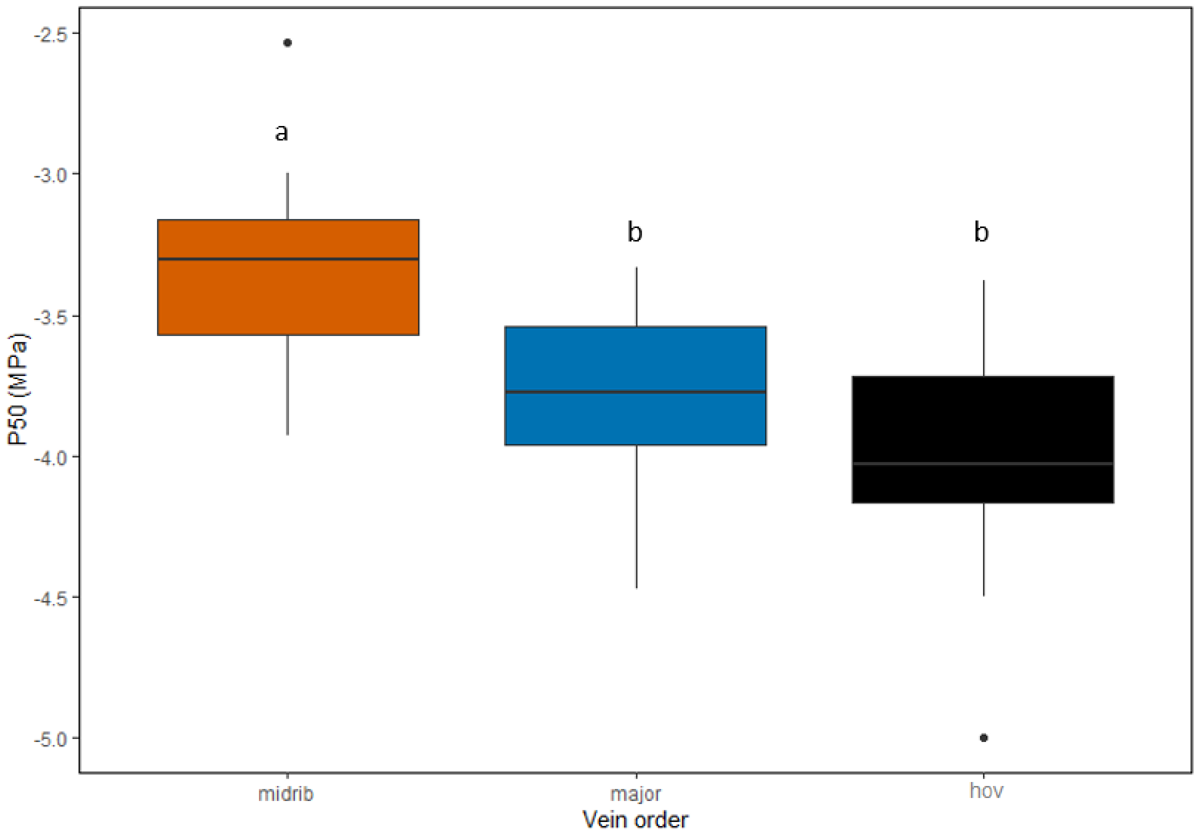

Supplemental Figure S6 Leaf water potential inducing a 50% loss of water transport capacity (P50, MPa) in the midrib, major and hov (high order veins) of *E. viminalis* leaves (n=13). Different letters indicate significant differences between orders (p-value <0.05). The horizontal line in boxes is the median value, and vertical lines are the 25th and 75th percentiles.

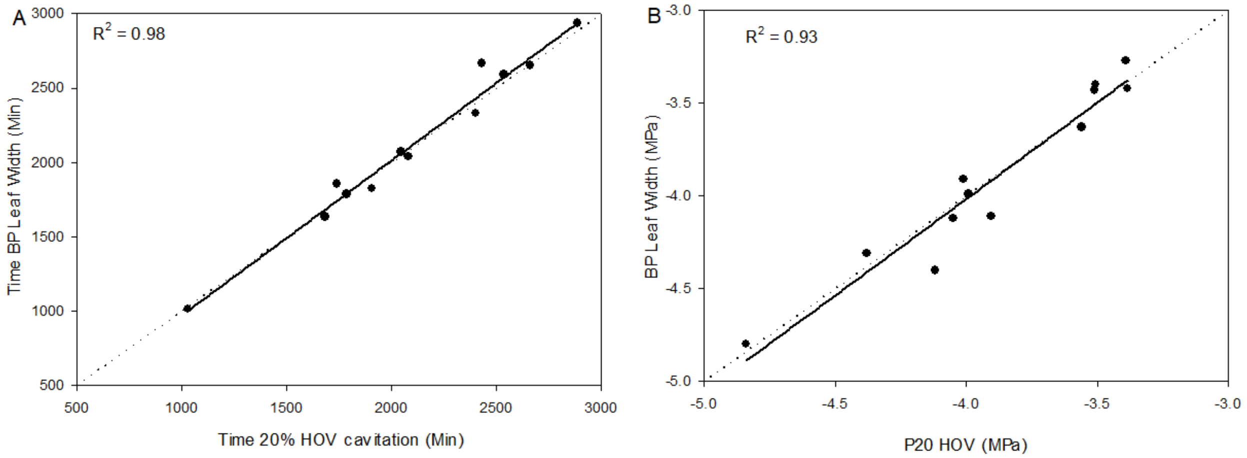

Supplemental Figure S7 The relationship between the breakpoint in leaf width and 20% HOV cavitation, suggesting a tight connection in time and water potential. (A) The relationship between the time at which we observed the breakpoint on leaf width to occur (Time BP Leaf width, Min) and the time at 20% high order vein cavitation (Time 20% HOV cavitation, Min) for each individual of *E. viminalis* (n=13) is described by a strong linear correlation (R^2 = 0.98, p-value <0.05). The distance of the residuals to the 1:1 relationship is -17.92 ± 88.96 Min.
(B) The relationship between the at which we observed the breakpoint on leaf width to occur (BP Leaf Width, MPa) and the Ψ_{stem} at 20% high order vein cavitation (P20 HOV, MPa) for each individual of E. viminalis (n=13) is described by a strong linear correlation (R^2 = 0.93, p-value <0.05). The distance of the residuals to the 1:1 relationship is 0.01 ± 0.13 MPa.
Dotted black lines indicate 1:1 relationship.

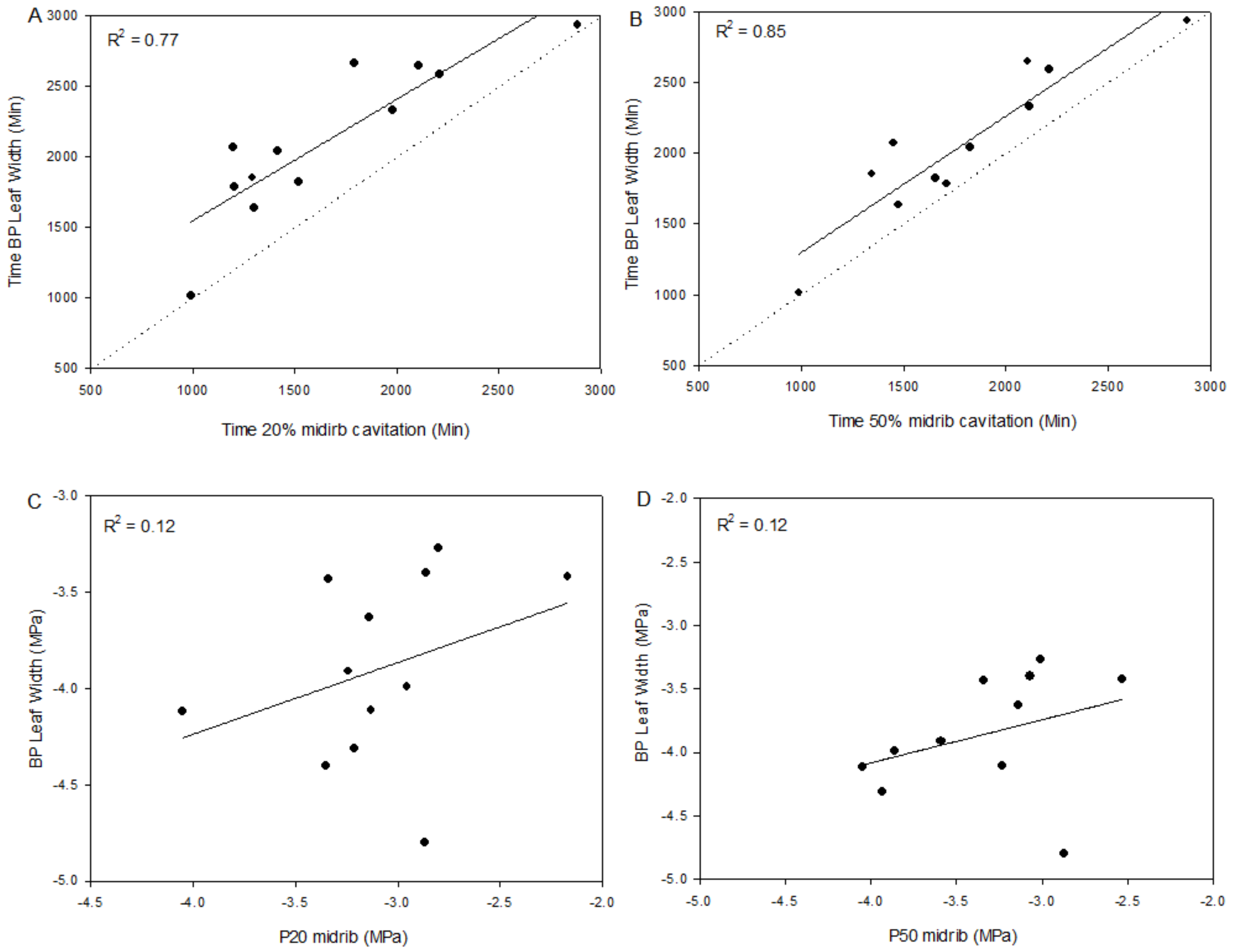

Supplemental Figure S8 Relationship between the time at which breakpoint in leaf width occur (BP Leaf Width, Min) and the time at which 20% of midrib cavitates (A) and the 50% of midrib cavitates (B), with the distance of the points to the 1:1 relationship been respectively -459.58 ± 261.57 Min and -269.55 ± 200.55 Min. Relationship between the Ψ_{stem} at which breakpoint in leaf width occur (BP Leaf Width, Min) and the Ψ_{stem} at which 20% of midrib cavitates (A) and the 50% of midrib cavitates (B). The distance of the residuals to the 1:1 relationship si respectively 0.79 ± 0.50 MPa and 0.54 ± 0.53 MPa.

R^2 values are reported and dotted black lines indicate 1:1 relationship.

Supplemental Method S1

Point of runaway vulnerability with full sigmoidal curve

Let our vulnerability curve be described using the sigmoidal function

$$K = \frac{K_{max}}{1+e^{(\phi - P_{50})/\alpha}}, \qquad (1)$$

where K is the hydraulic conductance (in mmol m^{-2} s^{-1} MPa^{-1}), K_{max} is the maximum hydraulic conductance (also in mmol m^{-2} s^{-1} MPa^{-1}), ϕ is the negative of water potential (so that water potential is represented by a positive quantity, in MPa), P_{50} is the ϕ where K is half maximum (also in MPa) and α is a width parameter (again, in MPa).

The liquid flux of water (J, in mol m^{-2} s^{-1}) is then given by

$$J = K(\phi - \phi_s) = \frac{K_{max}(\phi - \phi_s)}{1+e^{(\phi - P_{50})/\alpha}}, \qquad (2)$$

where ϕ_s is the negative of source water potential (in MPa).

Liquid and vapour fluxes of water are equal at steady state. If we consider the case of a constant cuticular transpiration (E_c, in mmol m^{-2} s^{-1}), then the steady state occurs when

$$J = E_c = \frac{K_{max}(\phi - \phi_s)}{1+e^{(\phi - P_{50})/\alpha}}. (3)$$

We are interested in finding the point of runaway vulnerability for the case of a constant cuticular transpiration (E_c, in mmol m^{-2} s^{-1}). The point of runaway vulnerability happens when J is a maximum (J_{max}) and is equal to E_c. This is easiest to see graphically by plotting J and E_c and observing where they intersect (the steady states). If $J_{max} > E_c$ then two steady states exist (one stable, one unstable), but the two solutions converge as either E_c or ϕ_s increases. At the point of runaway vulnerability, only one solution exists and $J_{max} = E_c$. If E_c or ϕ_s increases further, so that $J_{max} < E_c$, then no steady state exists and runaway vulnerability occurs. For one of E_c or ϕ_s given, there is a unique value for the other, and hence a value for ϕ, where runaway vulnerability occurs.

Now, J_{max} occurs when

$$\frac{dJ}{d\phi} = 0.$$

This gives

$$\frac{dJ}{d\phi} = K_{max}\left\{\frac{1}{1+e^{(\phi-P_{50})/\alpha}} - \frac{(\phi-\phi_s)}{\alpha}\frac{e^{(\phi-P_{50})/\alpha}}{\left[1+e^{(\phi-P_{50})/\alpha}\right]^2}\right\} = 0,$$

$$\frac{K_{max}}{\left[1+e^{(\phi-P_{50})/\alpha}\right]^2}\left\{1 + e^{(\phi-P_{50})/\alpha} - \frac{(\phi-\phi_s)}{\alpha}e^{(\phi-P_{50})/\alpha}\right\} = 0,$$

which can only equal zero if the term in the brackets is zero, so that

$$1 + e^{(\phi-P_{50})/\alpha} - \frac{(\phi-\phi_s)}{\alpha}e^{(\phi-P_{50})/\alpha} = 0. \qquad (4)$$

We can rearrange this equation to eliminate the exponential term, giving

$$\left[\frac{(\phi-\phi_s)}{\alpha} - 1\right]e^{(\phi-P_{50})/\alpha} = 1,$$

$$e^{(\phi-P_{50})/\alpha} = \frac{1}{\left[\frac{(\phi-\phi_s)}{\alpha}-1\right]},$$

$$1 + e^{(\phi-P_{50})/\alpha} = 1 + \frac{1}{\left[\frac{(\phi-\phi_s)}{\alpha}-1\right]},$$

$$1 + e^{(\phi-P_{50})/\alpha} = \frac{\frac{(\phi-\phi_s)}{\alpha}-1+1}{\left[\frac{(\phi-\phi_s)}{\alpha}-1\right]},$$

$$1 + e^{(\phi-P_{50})/\alpha} = \frac{\frac{(\phi-\phi_s)}{\alpha}}{\left[\frac{(\phi-\phi_s)}{\alpha}-1\right]},$$

$$\frac{1}{1+e^{(\phi-P_{50})/\alpha}} = \frac{\left[\frac{(\phi-\phi_s)}{\alpha}-1\right]}{\frac{(\phi-\phi_s)}{\alpha}}. \qquad (5)$$

Now, remember $J_{max} = E_c$ at the point of runaway cavitation. We can eliminate the exponential term in equation 3 using equation 5, which is true at J_{max}, giving

$$E_c = K_{max}(\phi - \phi_s)\frac{\left[\frac{(\phi-\phi_s)}{\alpha}-1\right]}{\frac{(\phi-\phi_s)}{\alpha}},$$

$$E_c = \alpha K_{max}\left[\frac{(\phi-\phi_s)}{\alpha} - 1\right],$$

$$\frac{E_c}{\alpha K_{max}} = \frac{(\phi-\phi_s)}{\alpha} - 1,$$

$$\phi - \phi_s - \alpha - \frac{E_c}{K_{max}} = 0. \qquad (6)$$

This equality holds at the point of runaway cavitation. We can eliminate one variable and substitute it back into equation 3 and solve for the other variable. As we want an equation for what ϕ runaway vulnerability occurs at for a given E_c, we will eliminate ϕ_s:

$$\phi_s = \phi - \alpha - \frac{E_c}{K_{max}}. \quad (7)$$

Substituting equation 7 again into equation 3 gives

$$E_c = \frac{K_{max}\left(\phi - \phi + \alpha + \frac{E_c}{K_{max}}\right)}{1 + e^{(\phi - P_{50})/\alpha}},$$

$$E_c = \frac{\alpha K_{max} + E_c}{1 + e^{(\phi - P_{50})/\alpha}},$$

$$1 + e^{(\phi - P_{50})/\alpha} = \frac{\alpha K_{max} + E_c}{E_c},$$

$$1 + e^{(\phi - P_{50})/\alpha} = 1 + \frac{\alpha K_{max}}{E_c},$$

$$e^{(\phi - P_{50})/\alpha} = \frac{\alpha K_{max}}{E_c},$$

$$(\phi - P_{50})/\alpha = \ln\left(\frac{\alpha K_{max}}{E_c}\right),$$

$$\phi = P_{50} + \alpha \ln\left(\frac{\alpha K_{max}}{E_c}\right). \qquad (8)$$

This gives the negative of water potential where runaway vulnerability will first occur.

We might also want to know what percentage loss of K this corresponds to. If we let the percentage loss of K be x, then

$$x = \left(1 - \frac{K}{K_{max}}\right) \times 100\%,$$

$$x = \left(1 - \frac{1}{1 + e^{(\phi - P_{50})/\alpha}}\right) \times 100\%,$$

$$x = \left(\frac{1 + e^{(\phi - P_{50})/\alpha} - 1}{1 + e^{(\phi - P_{50})/\alpha}}\right) \times 100\%,$$

$$x = \left(\frac{e^{(\phi - P_{50})/\alpha}}{1 + e^{(\phi - P_{50})/\alpha}}\right) \times 100\%.$$

Now, at runaway cavitation the exponential is

$$e^{(\phi - P_{50})/\alpha} = e^{\left(P_{50} + \alpha \ln\left(\frac{\alpha K_{max}}{E_c}\right) - P_{50}\right)/\alpha} = \frac{\alpha K_{max}}{E_c},$$

so that

$$x = \left(\frac{\frac{\alpha K_{max}}{E_c}}{1 + \frac{\alpha K_{max}}{E_c}}\right) \times 100\%. \qquad (9)$$

This gives the percentage loss of hydraulic conductance where runaway cavitation should occur.
